# P01 Who’s counting? How many IV antibiotic administrations are given daily at four Midlands trusts

**DOI:** 10.1093/jacamr/dlad143.005

**Published:** 2024-01-03

**Authors:** Abi Jenkins, Sadiya Hussain, Syed Gilani, Corrine Ashton

**Affiliations:** University Hospitals Birmingham NHS Foundation Trust, UK; Birmingham Women’s and Children’s NHS Foundation Trust, UK; Dudley Group NHS Foundation trust, UK; University Hospitals of Leicester, UK

## Abstract

**Background:**

Up to 80% of patients admitted to hospital receive IV therapy during their stay.^1^ In the UK, the NHS England Commissioning for Quality and Innovation (CQUIN) audit for quarter 1 of 2023–24 in the Midlands shows that 9.1%–35.0% of patients prescribed IV antibiotics are appropriate for a switch to oral therapy. A recent time and motion study demonstrated that IV medicine administration takes 20 min longer to give than an oral preparation.^2^ Consequently, an appropriate IV to oral switch releases 20 min of nurse time per dose administered. The unknown parameter is ‘how many antibiotic injections are given daily in hospitals’. Quantifying this parameter would enable Trusts to calculate the workforce capacity that could be realized on implementing an effective IVOS pathway.

**Methods:**

The number of IV antibiotics administered to inpatients was determined on one day for seven consecutive weeks at four Midlands trusts. In Week 1, data were collected on Sunday, Week 2 on Monday and so on until Week 7 on Saturday. Finally, the ratio of number of IV antibiotics doses per number of occupied beds was determined.

**Results:**

The range of the number of doses administered per occupied beds was between 0.92 and 1.18; it would be reasonable therefore to use the approximation of the number of antibiotic injections given daily at the four trusts as one injection per occupied bed. Taking a pragmatic approach that 20% of IV doses could be given orally with each change to oral releasing 20 min per administration we are proposing a calculation for the potential workforce released if an effective IVOS pathway is implemented: number of workforce hours released/day=number of trust beds/15.

**Conclusions:**

Implementation of an effective IVOS pathway has the potential to release a significant number of hours each day to nurses to undertake other tasks.

**Table 1.** Results for 7 day study period

Trust	No. of IV antibiotic doses given		No. of occupied beds		Total no. of doses/total no. of occupied beds
	Total	Per day, mean	Total	Per day, mean	
University Hospitals Birmingham	15110	2159	15872	2267	1.05
University Hospitals of Leicester	10825	1546	11808	1687	0.92
Dudley Group of Hospitals	5229	747	4431	633	1.18
Birmingham Women and Childrens	1837	262	1750	250	1.05

**Table 2.** Number of nursing hours released on implementation of IVOS pathway

Trust	Time released per day, h
University Hospitals Birmingham	151
University Hospitals of Leicester	112
Dudley Group of Hospitals	42
Birmingham Women and Childrens	16
